# Changes in antibiotic prescription following an education strategy for acute respiratory infections

**DOI:** 10.1038/s41533-021-00247-7

**Published:** 2021-06-03

**Authors:** Eloisa Delsors, Francisco Monsó, Francisco Javier López-Román, Juan Francisco Menárguez-Puche, María Gonzalez-Barberá, Hana Hukelova, Maria Teresa Martínez-Ros, Asensio López-Santiago

**Affiliations:** 1 Family Medicine, Primary Care Health Center Jesús Marín Molina de Segura, Murcia, Spain; 2 Pediatric Primary Care, Primary Care Health Center Jesús Marín, Murcia, Spain; 3 Biomedical Research Institute of Murcia (IMIB-Arrixaca), Murcia, Spain; 4 Health Sciences Department, Catholic University San Antonio (UCAM), Murcia, Spain; 5 Family Medicine, Primary Care Health Center Alcantarilla Sangonera, Murcia, Spain; 6 Dirección General de Planificación, Investigación, Farmacia y Atención al Ciudadano, Murcia, Spain; 7 Family Medicine, Dirección General de Planificación, Investigación, Farmacia y Atención al Ciudadano, Murcia, Spain; 8 Family Medicine, Servicio Murciano de Salud, Murcia, Spain

**Keywords:** Respiratory tract diseases, Therapeutics

## Abstract

The objective of this study was to assess the impact of an education intervention for primary health care physicians, based on the knowledge of clinical practice guidelines and availability of rapid antigen detection test for group A streptococci (GAS), on the improvement of antibiotic prescription for patients with acute respiratory tract infections. Before and after the intervention, physicians collected data from ten consecutive patients who attended during a 3-week period. This process was performed twice a year for 6 consecutive years (2012–2017). A total of 18,001 patients were visited by 391 primary care physicians during the study period, 55.6% before intervention and 44.4% after intervention. After intervention, the antibiotic prescription decreased significantly, from 33.0 to 23.4% (*p* < 0.01). However, there was a statistically significant increase (*p* < 0.01) in the use of penicillins. This study, carried out in daily practice conditions, confirms that the educational strategy was associated with an overall reduction in the use of antibiotics and an improvement in the antibiotic prescription profile in acute respiratory tract infections.

## Introduction

Infections are one of the most common reasons for consultations in primary care, accounting for approximately one-third of visits to the general practitioner, with half of these visits due to acute respiratory tract infections^1,2^. There is a large variability in the prescription of antibiotics between health care professionals and countries for the management of patients with acute respiratory tract infections. Inappropriate and excessive antibiotic consumption is the most important factor for the development of antibiotic resistance^3^. In countries with the highest antibiotic use, it is where more multiresistant pathogens are isolated^4^. Also, consumption of one antibiotic agent is associated with a greater propensity to resistance^5,6^. In addition, there has been an alarming decrease in the research of new antibiotic drugs^7,8^, with a clinical shortage of new molecules being approved in the past decades^9,10^. A few antibiotic classes in the pipeline are innovative treatments that will add value to the current antibiotic treatment arsenal.

Unnecessary prescribing of antibiotics is a major contributor to the problem of antimicrobial resistance, and quality improvement efforts appear generally effective at reducing both inappropriate use of antibiotics and choice of antibiotic drugs^11^. A high percentage of antibiotics are prescribed in the primary care setting, with acute lower respiratory tract illness as one of the most common condition^12^. The debate continues about the effect of antibiotics on the course of the disease, concerns regarding complications if antibiotics are not prescribed, or the definition of the clinical characteristics that identify patients at risk for which antibiotics are necessary. Single quality improvement antibiotic prescribing strategies (e.g., no offer or a delayed offer of antibiotics for acute uncomplicated lower respiratory tract infections, rapid antigen testing to detect group A Streptococcal [GAS] infection) have shown to be effective for the rational use of antibiotics^13,14^. Also, active clinician education may be more effective than passive education, particularly for addressing the antibiotic treatment decision^15^.

Therefore, the aim of this study was to assess changes in antibiotic prescription following an education intervention for primary health care physicians, based on the knowledge of clinical practice guidelines and availability of rapid antigen detection test for GAS, and on the improvement of antibiotic use for patients with acute respiratory tract infection.

## Results

### Participants

A total of 18,001 patients were visited by 391 primary care physicians during the study period, of whom 10,002 (55.6%) were attended before implementation of the educational intervention and the remaining 7999 (44.4%) in the post-intervention phase of the study. The number of physicians and patients included in each edition of the study is shown in Table 1. Fifty-eight percent of patients were women (before intervention 56%, after intervention 42%) and 42% were men (before intervention 55%, after intervention 45%). The mean (SD) age of the participants was 39.4 (15.7) years.

**Table 1:** Number of physicians and patients included in each edition of the study.

Years	Editions	Patients		Physicians
		Before	After	
2012	1st edition	2928	2839	101
	2nd edition	931	775	35
2013	1st edition	608	521	29
	2nd edition	734	448	31
2014	1st edition	638	551	25
	2nd edition	430	534	23
2015	1st edition	538	289	22
	2nd edition	592	422	25
2016	1st edition	536	569	29
	2nd edition	813	343	33
2017	1st edition	477	577	15
	2nd edition	777	131	23
Total		10,002	7999	391

As shown in Table 2, the most frequent symptoms were cough and/or rhinorrhea (72.8% of the cases), followed by painful swallowing (odynophagia) (50.8%) and fever (33.1%), with a similar distribution in the pre-intervention and post-intervention phases of the study.

**Table 2:** Changes of symptoms of acute respiratory tract infection before and after the physician’s educational intervention.

Symptoms	Total^a^, no. (%)	Educational intervention	
		Before^a^, no. (%)	After^a^, no. (%)
Fever	6021 (33.4)	3370 (33.7)	2651 (33.1)
Cough and/or rhinorrhea	13,106 (72.8)	7416 (74.1)	5690 (71.1)
Odynophagia	9581 (53.2)	5148 (51.5)	4433 (55.4)
Tonsillar exudate	1504 (8.4)	683 (6.8)	821 (10.3)
Purulent eye discharge	329 (1.8)	200 (2.0)	129 (1.6)
Painful cervical lymph nodes	1251 (6.9)	496 (5.0)	755 (9.4)
Dyspnea	1726 (9.6)	1015 (10.1)	711 (8.9)
Increased expectoration	3449 (19.2)	2001 (20.0)	1448 (18.1)
Purulent sputum	1416 (7.9)	835 (8.3)	581 (7.3)
None of the aforementioned	301 (1.7)	170 (1.7)	131 (1.6)

Total number of patients before the intervention was 10,002 and after the intervention was 7999.

^a^Frequency of symptoms is expressed as numbers and percentages in the overall study population and divided according to the time at which the patient was evaluated by the primary care physician, before or after the intervention.

### Results of the educational intervention

After the educational intervention, there was a statistically significant decrease in the prescription of antibiotics, from 33.0% (3221/9760) to 23.4% (1828/7812) (*p* < 0.01 (*χ*^2^)). In relation to the different pathological conditions (Table 3), the use of antibiotics decreased significantly when they were prescribed for acute otitis media (from 89.1% to 77.0%; *p* < 0.05 (*χ*^2^)) and chronic bronchitis (from 72.7 to 56.0%; *p* < 0.05 (*χ*^2^)). The use of antibiotics also showed an important decrease in cases of acute pharyngotonsillitis (from 44.4 to 32.7%) and exacerbation episodes of chronic obstructive pulmonary disease (COPD) (from 85.2 to 75.6%), although pre- and post-intervention differences were not statistically significant (*p* = 0.08 (*χ*^2^)).

**Table 3:** Changes in the use of antibiotics in the different pathological conditions before and after the physician’s educational intervention.

Pathological condition	Total^a^, no. (%)	Educational intervention		*p* Value (*χ*^2^)
		Before^a^, no. (%)	After^a^, no. (%)	
Common cold	262 (3.9)	201 (5.6)	61 (2.0)	0.183
Acute otitis media	283 (84.2)	179 (89.1)	104 (77.0)	0.022
Acute sinusitis	278 (85.8)	168 (86.2)	110 (85.3)	0.855
Acute pharyngitis and tonsillitis	2110 (39.0)	1290 (44.4)	820 (32.7)	0.089
Acute bronchitis	1055 (65.6)	689 (72.7)	366 (56.0)	0.013
Pneumonia	188 (89.1)	93 (89.4)	95 (88.8)	0.892
Exacerbation of COPD/chronic bronchitis	282 (80.8)	161 (85.2)	121 (75.6)	0.087
Influenza	89 (5.1)	70 (6.2)	19 (3.1)	0.297
Other conditions	213 (22.5)	153 (29.8)	60 (13.8)	0.006

^a^Frequency of antibiotic use is expressed as numbers and percentages in the overall study population and divided according to the time at which the patient was evaluated by the primary care physician, before or after the intervention.

Table 4 shows the classes of antibiotics prescribed for the different pathological conditions before and after the physician’s educational intervention. Overall, as compared with the pre-intervention phase, there was a significant decrease in the use of antibiotics in the post-intervention phase in common cold (5.5 vs. 2.0%; *p* < 0.01 (*χ*^2^)), acute otitis media (89.1 vs. 77.0%; *p* < 0.01 (*χ*^2^)), acute pharyngotonsillitis (44.0 vs. 32.7%; *p* < 0.01 (*χ*^2^)), acute bronchitis (72.7 vs. 56.0%; *p* < 0.01 (*χ*^2^)), acute exacerbation of COPD or chronic bronchitis (85.2 vs. 75.6%; *p* < 0.05 (*χ*^2^)), and influenza (6.2 vs. 3.1%; *p* < 0.01 (*χ*^2^)). Also, the use of penicillins increased significantly in the post-intervention phase for the indications of acute otitis media, acute sinusitis, acute pharyngotonsillitis, acute bronchitis, and pneumonia, whereas the use of amoxicillin/clavulanate decreases significantly for the same indications. In acute exacerbation episodes of COPD or chronic bronchitis, there was a significant decrease in the use of macrolides.

**Table 4:** Changes in the percentage of use of antibiotics for the different pathological conditions before and after the physician’s educational intervention.

Pathological conditions	Penicillins		Amoxicillin/clavulanate		Macrolides		Quinolones		Cephalosporins	
	Before (%)	After (%)	Before (%)	After (%)	Before (%)	After (%)	Before(%)	After (%)	Before (%)	After (%)
Common cold	0.7	0.3*	0.4	0.3	0.7	0.4	0.1	0.0	0.2	0.1
Acute otitis media	11.4	27.4**	39.3	25.2**	6.0	3.0	13.9	9.6	15.9	8.9
Acute sinusitis	7.7	31.8**	43.6	29.5**	7.7	7.8	10.8	7.0	14.9	7.0
Acute pharyngotonsillitis	14.1	19.5**	16.5	8.1**	9.2	2.7**	0.9	0.3**	2.3	0.5**
Acute bronchitis	7.8	16.4**	22.4	16.7**	17.7	9.9**	14.1	8.7**	7.9	3.8**
Pneumonia	4.8	18.7^**^	30.8	25.2	12.5	8.4	31.7	26.2	4.8	5.6
Exacerbation of COPD or chronic bronchitis	6.3	8.8	28.6	28.8	12.7	5.6*	27.0	23.1	7.9	2.5
Influenza	0.3	0.3	0.4	0.0	1.0	0.8	0.3	0.2	0.6	0.0

Data are expressed as the percentage of the total use of antibiotics for each pathological condition at the time at which the patient was evaluated by the primary care physician, before or after the intervention.

*P < 0.05; **p < 0.01 for the comparison before and after the intervention (*χ*^2^).

**Fig. 1:**
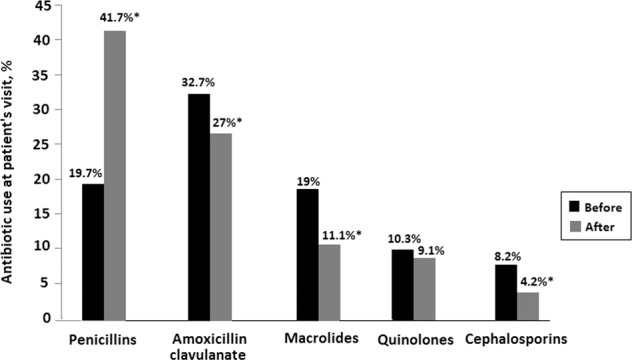
Percentage of use of the different antibiotic classes in relation to overall use of antibiotics before and after the educational intervention at the time at which the patient was evaluated by the primary care physician. *P < 0.01 for the comparison before and after the intervention (*χ*^2^).

In relation to the use of the different antibiotic classes before and after the intervention (Table 5), there was a statistically significant increase (*p* < 0.01 (*χ*^2^)) in the mean (SD) percentage of use of penicillins (19.7 [6.1] vs. 41.7% [9.4]) and significant reductions in the use of amoxicillin/clavulanate (32.7 [10.1] vs. 27.0% [6.1]), macrolides (19.0 [5.9] vs. 11.1% [2.5]), and cephalosporins (8.2 [2.5] vs. 4.2% [0.9]) (Fig. 1). Differences in the use of quinolones were not found.

**Table 5:** Changes in the percentage of use of the different antibiotic classes before and after the physician’s educational intervention for the different pathological conditions.

Antibiotic classes	Common cold	Acute otitis media	Acute sinusitis	Acute pharyngotonsillitis	Acute bronchitis	Pneumonia	COPD chronic bronchitis	Influenza
Penicillins (%)								
Before	4.4	3.9	2.5	69.1	12.4	0.8	2.0	0.5
After	1.4*	5.0**	5.6**	66.4**	14.6**	2.7**	1.9	0.3
Amoxicillin/clavulanate								
Before	1.4	8.0	8.6	48.5	21.4	3.2	5.5	0.5
After	1.7	7.2**	8.0^	43.0**	23.0**	5.7	9.7	0.0
Macrolides								
Before	4.2	2.1	2.6	46.6	29.2	2.3	4.2	1.9
After	6.1	2.0	5.1	34.7	33.2**	4.6	4.6*	2.6
Quinolones								
Before	0.6	9.0	6.8	8.4	43.2	10.6	16.5	1.0
After	0.6	8.1	5.6	4.4**	35.6**	17.5	23.1	0.6
Cephalosporins								
Before	2.8	13.0	11.8	27.6	30.5	2.0	6.1	2.8
After	2.7	16.2	12.2*	17.6**	33.8**	8.1	5.4*	0.0*

Data are expressed as the percentage of the total use of antibiotics for each pathological condition at the time at which the patient was evaluated by the primary care physician, before or after the intervention.

*P < 0.05; **p < 0.01 for the comparison before and after the intervention (*χ*^2^).

In relation to the rapid antigen testing for GAS at the time of consultation, of 2505 patients with acute pharyngotonsillitis, 1350 (53.9%) fulfilled the criteria for performing the test and underwent GAS testing. The test was positive in 520 cases (positivity rate 38.5%) and negative in the remaining 830. Antibiotics were prescribed in 98.1% of positive cases (510/520) and in 11.7% (97/839) of negative cases. The rate of antibiotic prescription for acute pharyngotonsillitis after the intervention was 32.7% as compared to 44.4% before the intervention (Table 3). In addition, there was a change in the antibiotic profile, with a decrease of amoxicillin/clavulanate, macrolides, quinolones, and cephalosporins, and an increase in penicillin (Table 4).

## Discussion

The main finding of this study is a significant reduction in prescribing antibiotic treatment (from 33.3 to 23.4%) by primary care physicians for patients with acute respiratory tract infections after implementation of an educational strategy, based on a better knowledge of clinical practice guidelines. Also, the profile of antibiotics prescribed was more in line with guidelines recommendations, with an overall increase of penicillins and a decrease of amoxicillin/clavulanate and macrolides.

The present results are consistent with previous studies in which educational interventions led to reduced antibiotic prescribing for respiratory tract infections. In a multi-faceted educational intervention in Norwegian general practice, general practitioners (*n* = 382) received two visits by peer academic detailers, the first presenting the national clinical guidelines for antibiotic use and recent research evidence on acute respiratory tract infections, and the second based on feedback reports on each general practitioner’s antibiotic prescribing profile from the preceding year^16^. There was a reduction in prescribing of antibiotics for acute respiratory tract infections (odds ratio 0.72, 95% confidence interval 0.61–0.84) and for prescribing non-penicillin antibiotics when an antibiotic was issued^16^. In a study that assessed views of experts from five countries who develop guidelines and interventions for respiratory tract infections, a consistent set of recommendations included to address general practitioner concerns about recommendations and explain the need for guidelines, to provide interventions that engage physicians, and to provide consistent educational messages about antibiotic use^17^. In a systematic review of 39 studies assessing the effectiveness of different educational interventions (printed materials, audit and feedback, physician reminders, educational meetings, outreach visits), all interventions reduced the use of antibiotics prescribed by health care professionals in the outpatient setting, although effectiveness depends to a large extent on the particular prescribing behavior and the barriers to change in the particular community^18^.

There are difficulties in clinical practice to distinguish bacterial from viral respiratory tract infections. Although the criteria for the use of antibiotics in COPD exacerbations have been established by Anthonisen et al.^19^, which comprise increased dyspnea, increased sputum volume, and increased sputum purulence, or Centor criteria (lymphadenectomy, no cough, fever, tonsillar exudate)^20^ for acute pharyngotonsillitis, antibiotic overuse is a widespread practice and a key driver of antibiotic resistance. In the present study, before implementation of the educational strategy, 85.2% of patients with acute exacerbation of COPD received antibiotics, which is >81.9% reported in a study of 332 Spanish general practitioners who received guidelines and participated in discussion sessions^21^. In patients with pharyngotonsillitis, the percentage of antibiotic prescription of 44.4% was <49.1% found in an observational study with the participation of 126 general practitioners from eight autonomous communities in Spain^22^. The availability of the rapid antigen testing for GAS at the time of consultation also promoted a reduction in antibiotic prescribing as shown by others^23^. In other pathological conditions, such as acute bronchitis and acute otitis media, there was a significant reduction in prescribing antibiotics after the physician’s educational intervention. The percentage of antibiotic use in the pre-intervention phase in acute bronchitis was 72%, which is higher than 62.3% found in a similar study^21^, and decreasing to 56% after the intervention.

Another effect observed after the educational intervention has been a change in the prescribing antibiotic profile, with a clear decrease in the use of broad-spectrum macrolides and amoxicillin/clavulanate and an increase in the prescription of narrow-spectrum antibiotics (penicillin). These data are consistent with the WHO recommendations in its classification of antibiotics^24^. The ability of professionals to introduce changes in their clinical practice after an educational intervention has been documented in other studies^18^. The correlation of behavioral changes with antibiotic prescription practices may potentially result in a reduction of antibiotic resistance as antibiotic overuse is the principal cause of emergence of resistance^3^. The reduction in the use of broad-spectrum antibiotics may also contribute to reducing bacterial resistance of pathogens usually involved in acute respiratory tract infections^11,17,25^. In a retrospective observational study including all episodes of respiratory tract infections registered during a 1-year period in a north-eastern Spanish region, a high prescription of broad-spectrum agents and antibiotics not recommended as the first choice was observed, with a high risk of increasing antimicrobial resistance^26^.

Nevertheless, this study also has certain limitations. As this was a retrospective study with voluntary participation of physicians, the potential for selection bias and confounding cannot be excluded. Moreover, this study was conducted only in Spanish patients, and it is, therefore, uncertain whether these findings can be extrapolated to a more diverse group of patients and to other health care systems. Although the large study population and the repeated educational interventions along the years consistently showing a benefit of the intervention to a more rational antibiotic prescription in acute respiratory tract infections, the lack of long-term follow-up, and the possibility for a waning off of the benefit of the intervention should be also taken into account as a limitation of the study. The study was designed as 10-day short-term periods before and after the intervention, and was repeated twice a year over various consecutive years. The consistency of findings in the reduction of antibiotic prescription in the different editions from 2012 to 2017 following this study design reinforces the impact of an educational intervention combined with GAS testing to sensitize primary care physicians on the better prescription of antibiotics for acute respiratory tract infections. However, other factors that might have influenced antibiotics prescribing over this time cannot be excluded.

In conclusion, this study carried out in daily practice conditions based on an educational strategy, with advice on the implementation of recommendations of clinical practice guidelines and the use of rapid antigen testing for GAS at the time of consultation in acute pharyngotonsillitis, shows an overall reduction of the use of antibiotics and an improvement in the antibiotic prescribing profile of primary care physicians for patients with acute respiratory tract infections. However, the amount of antibiotics prescribed in primary care is an important issue that needs exploration in further studies.

## Methods

### Study design

This was a retrospective observational study using data collected from an anonymized database that belonged to subjects who were previously involved in the HAPPY AUDIT project (acronym of Health Alliance for Prudent Prescribing, Yield And Use of Antimicrobial Drugs In the Treatment of Respiratory Tract Infection). In such a project, the primary objective was to assess the impact of an education strategy, based on the therapeutic adequacy of the use of antibiotics in respiratory infections combined with the use of rapid antigen testing for GAS, on changes in antibiotic prescription for acute respiratory tract infection. The study was conducted in the primary health care setting in the Region of Murcia, Spain (an autonomous community in the southeast on the Mediterranean coast, with a population of 1.4 million) between 2012 and 2017. The study protocol was approved by the Ethics Committee for Clinical Research of Hospital Virgen de la Arrixaca, Murcia, Spain. Written consent was waived because data used in this study were extracted from an anonymous database.

### Patients

The study population consisted of male and female patients aged >14 years who had received a physician’s diagnosis of acute respiratory tract infection (ICD-10) in the last month and who had not received antibiotics in the previous month prior to their inclusion in the study. Patients with severe or terminal illness were excluded.

### Study procedures

Primary care physicians were invited to participate in the study, which included a systematic registration of patients with acute respiratory tract infection attended in their daily practices during 10 consecutive days before and after the intervention. Registration of patients was carried out by the attending physicians who participated in the study. This first phase of the study was followed by the intervention, which was an accredited education session. This included a 4-h face-to-face course divided into two parts. In the first part, participants were instructed regarding recommendations of current clinical practice guidelines for the management of respiratory tract infections and the use of antibiotics. In the second part, they were instructed regarding the use of rapid antigen testing for GAS in patients with acute pharyngotonsillitis, with information on technical aspects of the test, indications (patients with odynophagia and in the presence of at least two of the following criteria: fever >38 °C, anterior cervical lymphadenopathy, and tonsillar exudate), and interpretation. In addition to theoretical training, participants performed an exercise for the acquisition of practical skills. The key characteristics of the GAS test include a time needed for results of ~10 min, sensitivity 95%, specificity 93%, positive predictive value 79.2%, and negative predictive value 98.5%.

After the education sessions, participants performed a second 10-day registration of consecutive patients with acute respiratory tract infection attended in daily practice. Also, they have available the rapid antigen testing for GAS in their consultation. This three-step process (pre-intervention patients’ registration, intervention, post-intervention patients’ registration) was performed twice a year (March–April–May and November–October–December) over 6 consecutive years (2012–2017). Clinicians and patients involved at different time points were not the same persons, that is, both clinicians and patients participated only once.

### Data collection

Data were recorded according to the methodology of HAPPY AUDIT project^27^ using the Audit Project Odense method^28,29^.

During the pre-intervention and post-intervention phases of the study, the following data were collected: demographics (age, sex); duration of symptoms (number of days); symptoms (including fever, cough, rhinorrhea, purulent ear discharge, painful swallowing; tonsillar exudate, painful cervical lymph nodes, dyspnea, increase expectoration, purulent sputum, and none of the aforementioned); rapid antigen testing for GAS (positive, negative, not performed); chest radiography (positive, negative, not performed); etiology of infection (probably viral, probably bacterial); and diagnosis according to ICD-10 codes in primary care, including common cold (code J00), acute otitis media (code H66.9), acute sinusitis (code J01), acute pharyngitis (code J02.9), unspecified acute tonsillitis (code J03.90), acute bronchitis (code J20), pneumonia (codes J12, J13, J14, J15, J16, J17, J18), exacerbation of COPD or chronic bronchitis (codes J41, J42, J43, J44), influenza (code J11.1), and other infections of the respiratory tract (code J06,9). Use or no use of antibiotics and the class of antibiotics administered (penicillins, amoxicillin, amoxicillin/clavulanate, macrolides, quinolones, tetracyclines, cephalosporins, and others) were recorded. Other variables were the presence of penicillin allergy, patient’s demand for antibiotic treatment, and patient’s referral to the hospital or to a specialist.

### Statistical analysis

Categorical variables are expressed as frequencies and percentages, and continuous variables as mean and standard deviation. The *χ*^2^ test was used for the comparison of categorical variables, and the Student’s *t* test was used for continuous data. Statistical significance was set at *p* < 0.05. The SPSS statistical program version 18.0 was used for statistical analysis.

### Reporting summary

Further information on research design is available in the Nature Research Reporting Summary linked to this article.

## Supplementary information

Reporting Summary

## Data Availability

Data are available from the corresponding author upon request.
