# Supplementary material for: Changes in antibiotic prescription following an education strategy for acute respiratory infections
Source: NPJ Prim Care Respir Med. 2021 Jun 3;31:34. doi: 10.1038/s41533-021-00247-7 (PMC8175562; doi:10.1038/s41533-021-00247-7)
Supplement: Supplementary file 1 — Reporting Summary [file 41533_2021_247_MOESM1_ESM.pdf]

nature research

Corresponding author(s): Juan Francisco Menarguez Puche

Last updated by author(s): Jul 15, 2020

Reporting Summary

Nature Research wishes to improve the reproducibility of the work that we publish. This form provides structure for consistency and transparency in reporting. For further information on Nature Research policies, see our Editorial Policies and the Editorial Policy Checklist.

Statistics

For all statistical analyses, confirm that the following items are present in the figure legend, table legend, main text, or Methods section.

n/a	Confirmed	
☒	☐	The exact sample size (n) for each experimental group/condition, given as a discrete number and unit of measurement
☐	☒	A statement on whether measurements were taken from distinct samples or whether the same sample was measured repeatedly
☐	☒	The statistical test(s) used AND whether they are one- or two-sided<br>*Only common tests should be described solely by name; describe more complex techniques in the Methods section.*
☒	☐	A description of all covariates tested
☐	☒	A description of any assumptions or corrections, such as tests of normality and adjustment for multiple comparisons
☐	☒	A full description of the statistical parameters including central tendency (e.g. means) or other basic estimates (e.g. regression coefficient) AND variation (e.g. standard deviation) or associated estimates of uncertainty (e.g. confidence intervals)
☐	☒	For null hypothesis testing, the test statistic (e.g. F, t, r) with confidence intervals, effect sizes, degrees of freedom and P value noted<br>*Give P values as exact values whenever suitable.*
☒	☐	For Bayesian analysis, information on the choice of priors and Markov chain Monte Carlo settings
☒	☐	For hierarchical and complex designs, identification of the appropriate level for tests and full reporting of outcomes
☒	☐	Estimates of effect sizes (e.g. Cohen's d, Pearson's r), indicating how they were calculated

Our web collection on statistics for biologists contains articles on many of the points above.

Software and code

Policy information about availability of computer code

Data collection	n/a
Data analysis	n/a

For manuscripts utilizing custom algorithms or software that are central to the research but not yet described in published literature, software must be made available to editors and reviewers. We strongly encourage code deposition in a community repository (e.g. GitHub). See the Nature Research guidelines for submitting code & software for further information.

Data

Policy information about availability of data

All manuscripts must include a data availability statement. This statement should provide the following information, where applicable:

- Accession codes, unique identifiers, or web links for publicly available datasets
- A list of figures that have associated raw data
- A description of any restrictions on data availability

The manuscripts include a data availability statement

Field-specific reporting

Please select the one below that is the best fit for your research. If you are not sure, read the appropriate sections before making your selection.

☒ Life sciences ☐ Behavioural & social sciences ☐ Ecological, evolutionary & environmental sciences

For a reference copy of the document with all sections, see nature.com/documents/nr-reporting-summary-flat.pdf

Life sciences study design

All studies must disclose on these points even when the disclosure is negative.

Sample size	All subjects whose data were collected in the database were included in the study.
Data exclusions	No data were excluded from the analysis
Replication	Data were extracted from a database that were constructed over the work of 6 years and therefore findings cannot be reproduced easily because of the lack of experimental findings in a single experiment.
Randomization	n/a
Blinding	n/a

Reporting for specific materials, systems and methods

We require information from authors about some types of materials, experimental systems and methods used in many studies. Here, indicate whether each material, system or method listed is relevant to your study. If you are not sure if a list item applies to your research, read the appropriate section before selecting a response.

Materials & experimental systems

n/a	Involved in the study	
☒	☐	Antibodies
☒	☐	Eukaryotic cell lines
☒	☐	Palaeontology and archaeology
☒	☐	Animals and other organisms
☐	☒	Human research participants
☐	☒	Clinical data
☒	☐	Dual use research of concern

Methods

n/a	Involved in the study	
☒	☐	ChIP-seq
☒	☐	Flow cytometry
☒	☐	MRI-based neuroimaging

Human research participants

Policy information about studies involving human research participants

Population characteristics	The study population consisted of male and female patients aged > 14 years who had received a physician's diagnosis of acute respiratory tract infection (ICD-10) in the last month and who had not received antibiotics in the previous month prior to their inclusion in the study. Patients with severe or terminal illness were excluded.
Recruitment	This was a retrospective observational study using data collected from an anonymized database belonged to subjects that were previously involved in the HAPPY AUDIT project
Ethics oversight	The study protocol was approved by the Ethics Committee for Clinical Research of Hospital Virgen de la Arrixaca, Murcia, Spain

Note that full information on the approval of the study protocol must also be provided in the manuscript.

Clinical data

Policy information about clinical studies

All manuscripts should comply with the ICMJE guidelines for publication of clinical research and a completed CONSORT checklist must be included with all submissions.

Clinical trial registration	n/a
Study protocol	n/a

Data collection	The data were collected from an anonymized database belonged to subjects that were previously involved in the HAPPY AUDIT project
Outcomes	During the pre-intervention and post-intervention phases of the study, the following data were collected: demographics (age, sex); duration of symptoms (number of days); symptoms (including fever, cough, rhinorrhea, purulent ear discharge, painful swallowing; tonsillar exudate, painful cervical lymph nodes, dyspnea, increase expectoration, purulent sputum, and none of the aforementioned); rapid antigen testing for GAS (positive, negative, not performed); chest radiography (positive, negative, not performed); etiology of infection (probably viral, probably bacterial); and diagnosis according to ICD-10 codes in primary care, including common cold (code J00), acute otitis media (code H66.9), acute sinusitis (code J01), acute pharyngitis (code J02.9), unspecified acute tonsillitis (code J03.90), acute bronchitis (code J20), pneumonia (codes J12, J13, J14, J15, J16, J17, J18), exacerbation of chronic obstructive pulmonary disease (COPD) or chronic bronchitis (codes J41, J42, J43, J44), influenza (code J11.1), and other infections of the respiratory tract (code J06,9). Use or not use of antibiotics and the class of antibiotics administered (penicillins, amoxicillin, amoxicillin/clavulanate, macrolides, quinolones, tetracyclines, cephalosporins, and others) were recorded. Other variables were the presence of penicillin allergy, patient's demand of antibiotic treatment, and patient's referral to the hospital or to a specialist.
